# Recurrent hotspot SF3B1 mutations at codon 625 in vulvovaginal mucosal melanoma identified in a study of 27 Australian mucosal melanomas

**DOI:** 10.18632/oncotarget.26584

**Published:** 2019-01-29

**Authors:** Camelia Quek, Robert V. Rawson, Peter M. Ferguson, Ping Shang, Ines Silva, Robyn P.M. Saw, Kerwin Shannon, John F. Thompson, Nicholas K. Hayward, Georgina V. Long, Graham J. Mann, Richard A. Scolyer, James S. Wilmott

**Affiliations:** ^1^ Melanoma Institute Australia, The University of Sydney, Sydney, Australia; ^2^ Sydney Medical School, The University of Sydney, Sydney, Australia; ^3^ Royal Prince Alfred Hospital, Sydney, Australia; ^4^ Royal North Shore Hospital, Sydney, Australia; ^5^ Centre for Cancer Research, Westmead Institute for Medical Research, Sydney, Australia; ^6^ QIMR Berghofer Medical Research Institute, Brisbane, Australia

**Keywords:** spliceosome, mucosal melanoma, SF3B1, hotspot mutation, targeted sequencing

## Abstract

**Introduction:**

Clinical outcomes for mucosal melanomas are often poor due to a lack of effective systemic drug therapies. Identifying driver genes in mucosal melanoma may enhance the understanding of disease pathogenesis and provide novel opportunities to develop effective therapies.

**Results:**

Somatic variant analysis identified *SF3B1* (6 of 27: 22%) as the most commonly mutated gene, followed by *KIT* (3 of 27: 11%). Other less frequently mutated genes (4% otherwise stated) included *BRAF* (7%), *NRAS* (7%), *ARID2*, *CTNNB1*, *DICER1*, *MAP2K1*, *NF1*, *PTEN*, *SETD2* and *TP53*. Recurrent SF3B1 p.R625 hotspot mutations were exclusively detected in vulvovaginal (5 of 19: 26%) and anorectal melanomas (3 of 5:60%). The only other SF3B1 mutation was a p.C1123Y mutation that occurred in a conjunctival mucosal melanoma.

*SF3B1*-mutated patients were associated with shorter overall survival (OS; 34.9 months) and progression-free survival (PFS; 16.9 months) compared to non-*SF3B1*-mutated patients (OS: 79.7 months, log-rank *P* = 0.1172; PFS: 35.7 months, log-rank *P* = 0.0963).

**Conclusion:**

Molecular subgroups of mucosal melanoma with *SF3B1* mutations occurred predominantly in the vulvovaginal region. *SF3B1* mutations may have a negative prognostic impact.

**Methods:**

Formalin-fixed biopsies were collected from 27 pathologically-confirmed mucosal melanomas. Genomic DNA was isolated from the tumor tissue and sequenced using a novel dual-strand amplicon sequencing technique to determine the frequency and types of mutations across 45 target genes.

## INTRODUCTION

Mucosal melanoma is a rare subtype of melanoma that originates from melanocytes in the epithelial lining of the conjunctiva, respiratory, alimentary, and genitourinary tracts. Although a majority of mucosal melanomas arise from the mucosa of the nasal cavity, oral cavity, anorectum, vulvovaginal, they can arise in any part of mucosal membranes. Mucosal melanomas comprise approximately 1% of all melanomas in European populations, but up to 25% in Asian populations [1]. Because mucosal melanoma occurs at sites that are not easily amenable to clinical inspection, patients with mucosal melanoma frequently present with advanced-stage disease often with regional and/or distant metastases, and their prognosis is generally poor [2, 3]. In contrast to cutaneous melanomas that are typically associated with exogenous (ultraviolet light exposure) and endogenous (genetic predisposition) risk factors [4], there are no clear risk factors for mucosal melanomas, and the molecular pathogenesis of mucosal melanoma is not well defined.

Several studies have shown that mucosal melanomas have distinct molecular profiles [5–11]. Molecular drivers such as BRAF p.V600 mutations that are amenable to therapeutic intervention (with a combination of BRAF and MEK inhibitors) are uncommon in mucosal melanomas (<10% of cases) as compared to cutaneous melanoma, where approximately 40% are BRAF mutant; of these 74% are the V600E genotype and 22% are V600K [5–9]. Mutations in *NRAS* only occur in 10–20% of mucosal melanomas [5, 6], while mutations in *GNAQ* and *GNA11* that are commonly detected in uveal melanoma, occur in approximately 9.5% of mucosal melanomas [10, 11]. Other activating oncogenic events, including the gain-of-function mutations of *KIT,* are present in approximately 15% of mucosal melanomas [10, 11] but are rare in cutaneous melanomas [12–14]. Unfortunately, the oncogenic drivers of mucosal melanoma remain poorly defined, nor it is known whether they vary in prevalence among melanomas from different mucosal sites of the body.

Recently, we performed whole-genome sequencing (WGS) on a large cohort (*n* =183) of cutaneous (*n* = 140), acral (*n* = 35) and mucosal melanomas (*n* = 8), and *SF3B1* (splicing factor 3B subunit 1) was identified as significantly mutated gene in mucosal melanoma [15]. *SF3B1* mutations are the most common spliceosomal component gene mutation implicated in the pathogenesis of cancer and act by causing aberrant RNA splicing events [16–19]. Among the different subtypes of melanoma, deleterious somatic variants in *SF3B1* were identified in 20% of uveal melanomas [17, 20, 21]. Several studies have identified mutations in *SF3B1* in subsets of solid tumors, as well as in myelodysplastic syndrome and chronic lymphocytic leukemia (CLL), in which they occurred in almost 15% of the reported cases [18, 22].

Common molecular drivers and mutations affecting spliceosomal components such as *SF3B1* have been reported to be associated with disease outcome in some cancer types, but not in mucosal melanoma [20, 23–26]. In this study, we sought to determine the prevalence of genetic alterations in *SF3B1* and of common oncogenic driver genes in mucosal melanomas, and investigate their impact on clinicopathologic characteristics and patient outcomes. To do this, we performed a novel dual-strand amplicon-based targeted sequencing covering all the previously defined significantly mutated melanoma genes [15] in a cohort of 27 mucosal melanomas arising from a variety of anatomical locations including vulvovaginal, anorectal, nasopharyngeal, conjunctival and oropharyngeal sites.

## RESULTS

### Mucosal melanoma patient characteristics

There were 27 patients included in this study (Table 1 and Supplementary Table 1); 22 females (81%) and 5 males (19%), with a median age at diagnosis of 65.5 years (range 29 to 109 years). The primary melanomas were located in the vulva/vagina (*n* = 15, 55%), anorectal region (*n* = 5, 18.5%), nasopharynx (*n* = 5, 18.5%), conjunctiva (*n* = 1, 4%) and palate (*n* = 1, 4%). Fourteen (52%) patients had T4 disease, three (11%) had T3 disease, and the remaining patients had T0, T1 or T2 disease. Tumor thickness was >1 mm in 22 (82%) patients. Median mitotic rate was 15 mitoses/mm^2^ and ulceration was present in 16 of 22 patients (72%) with known ulceration status.

**Table 1:** Clinicopathological characteristics of patients with mucosal melanoma (*n* = 27)

Characteristics	*N* (%)^a^
Age (median, IQR)	61 years, 51–78 years
Gender Female Male	22 (81%) 5 (19%)
Site Vulva Vagina Rectum Anus Conjunctiva Nasal cavity Nasal Sinus Palate	12 (44%) 3 (11%) 3 (11%) 2 (7%) 1 (4%) 4 (15%) 1 (4%) 1 (4%)
T classification 0 1 2 3 4 Data unavailable	1 (4%) 1 (4%) 5 (19%) 3 (11%) 14 (52%) 3 (11%)
N classification N0 N+ Data unavailable	16 (59%) 7 (26%) 4 (15%)
Mitotic rate (number of mitoses/mm^2^) < 10 ≥ 10 Data unavailable	7 (26%) 14 (52%) 6 (22%)
Ulceration Absent Present Data unavailable	6 (22%) 16 (59%) 5 (19%)
Tumor thickness (mm) <1 1–4 ≥ 4 Data unavailable	1 (4%) 8 (30%) 14 (52%) 4 (15%)

^a^ Unless otherwise indicated.

IQR indicates interquartile range.

### *SF3B1* and *KIT* mutations are oncogenic driver mutations in mucosal melanoma

**Figure 1:**
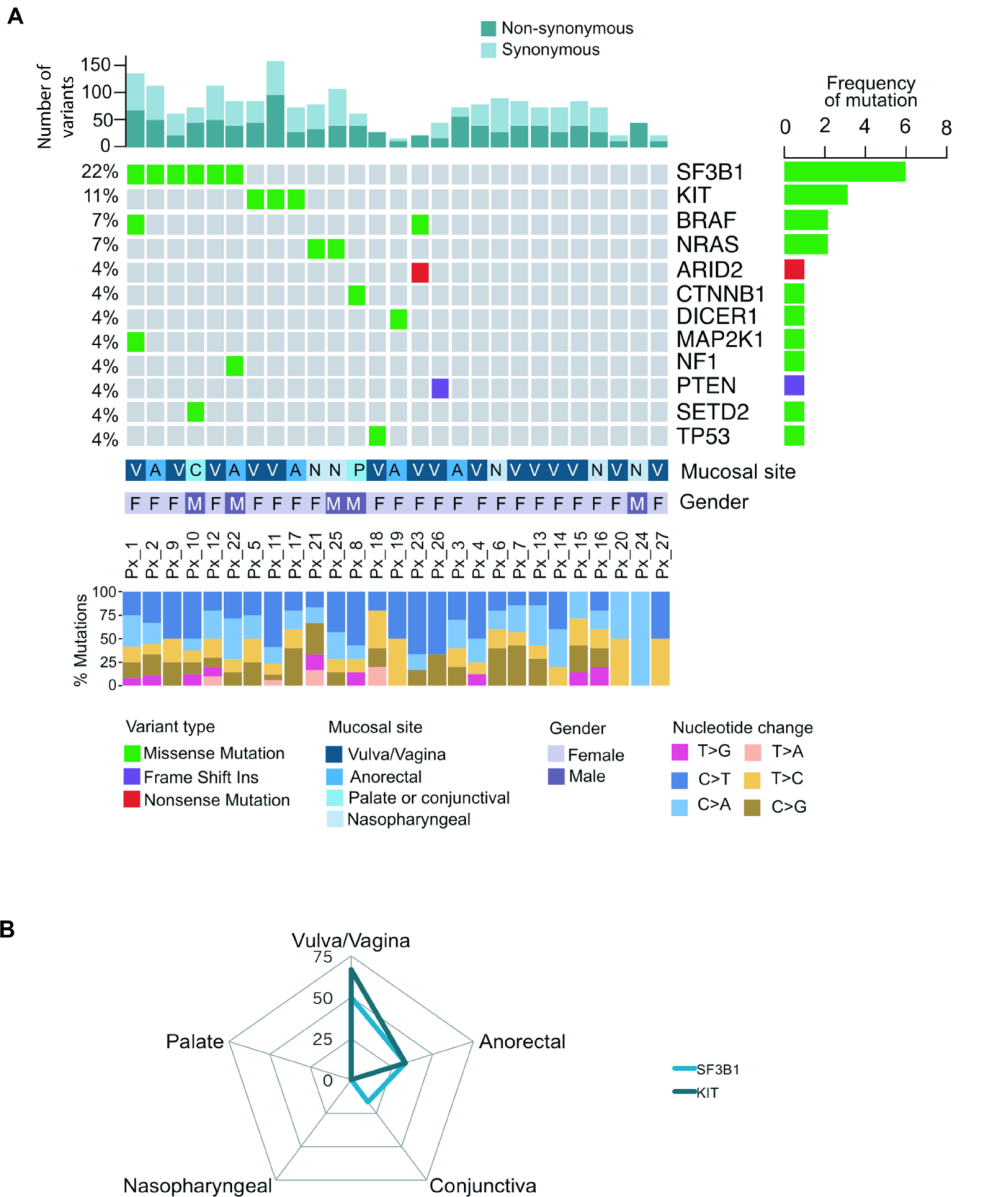
Mutational landscape of 27 melanoma patients across different mucosal sites (**A**) The oncoplot showing the distribution of different mutational types across 19 genes. Each column represents a mucosal melanoma sample (individual patient). Number of driver events for each patient is shown at the top of the panel. The frequency of mutation for each gene is shown in the right panel. The mucosal site, gender and percentage of nucleotide changes for individual patients are presented in the lower panel. (**B**) The radar graph displays the frequency of various gene mutations in different mucosal sites including vulva/vagina, anorectal, nasopharyngeal, conjunctiva and palate. Height of the peak indicates the frequency of individuals with a mutation in the respective mucosal site.

A 45 gene targeted NGS panel was designed to include all significantly mutated genes (SMG) identified in cutaneous, mucosal or acral melanomas in our previous publications [15, 27]. The panel includes all SMG identified in the Hayward *et al* or TCGA SKCM datasets that were detected using the MutSig, OncodriveFML or IntOGen driver detection tools for coding mutations and OncodriveFML for non-coding genes, as outlined in the prior studies [15, 27, 28]. The targeted NGS panel identified a total of 1435 variants that passed the variant caller filters with a median coverage depth of 2,700X (1,000–22,113X). The NGS panel identified *SF3B1* (6 of 27: 22%) as the most commonly mutated gene, followed by *KIT* (3 of 27: 11%) (Figure 1A). Other less frequently mutated genes included *BRAF* (7%), *NRAS* (7%), *ARID2*, *CTNNB1*, *DICER1*, *MAP2K1*, *NF1*, *PTEN*, *SETD2* and *TP53* (all 4%, Figure 1A). Both *SF3B1* (50%) and *KIT* (67%) mutations were most frequently mutated in tumors of female genital origin and anorectal region (33.3% for *SF3B1* and *KIT*) compared to a single *SF3B1* mutant conjunctival melanoma in the upper body sites (Figure 1B). All five *SF3B1* mutations (5 of 6: 83%) that occurred in the known hotspot p.R625H/L originated in the anorectal or female genital region, while the conjunctival primary harbored a *SF3B1* p.C1123Y mutation (Figure 1B). The *KIT* mutations were all missense, and of these, two were the hotspot mutation p.L576P in exon 11, and the other a p.T670I mutation in exon 14. *KIT* mutations in exon 11 and 14 are known to occur in thymic cancer and cutaneous melanoma, and gastrointestinal stromal tumors, respectively. The details of the oncogenic classification (oncodriveMUT), nucleotide and amino acid changes are summarized in Supplementary Table 2.

Recurrent hotspot *SF3B1* mutations at codon 625 in anorectal and vulva/vaginal melanomas

**Figure 2:**
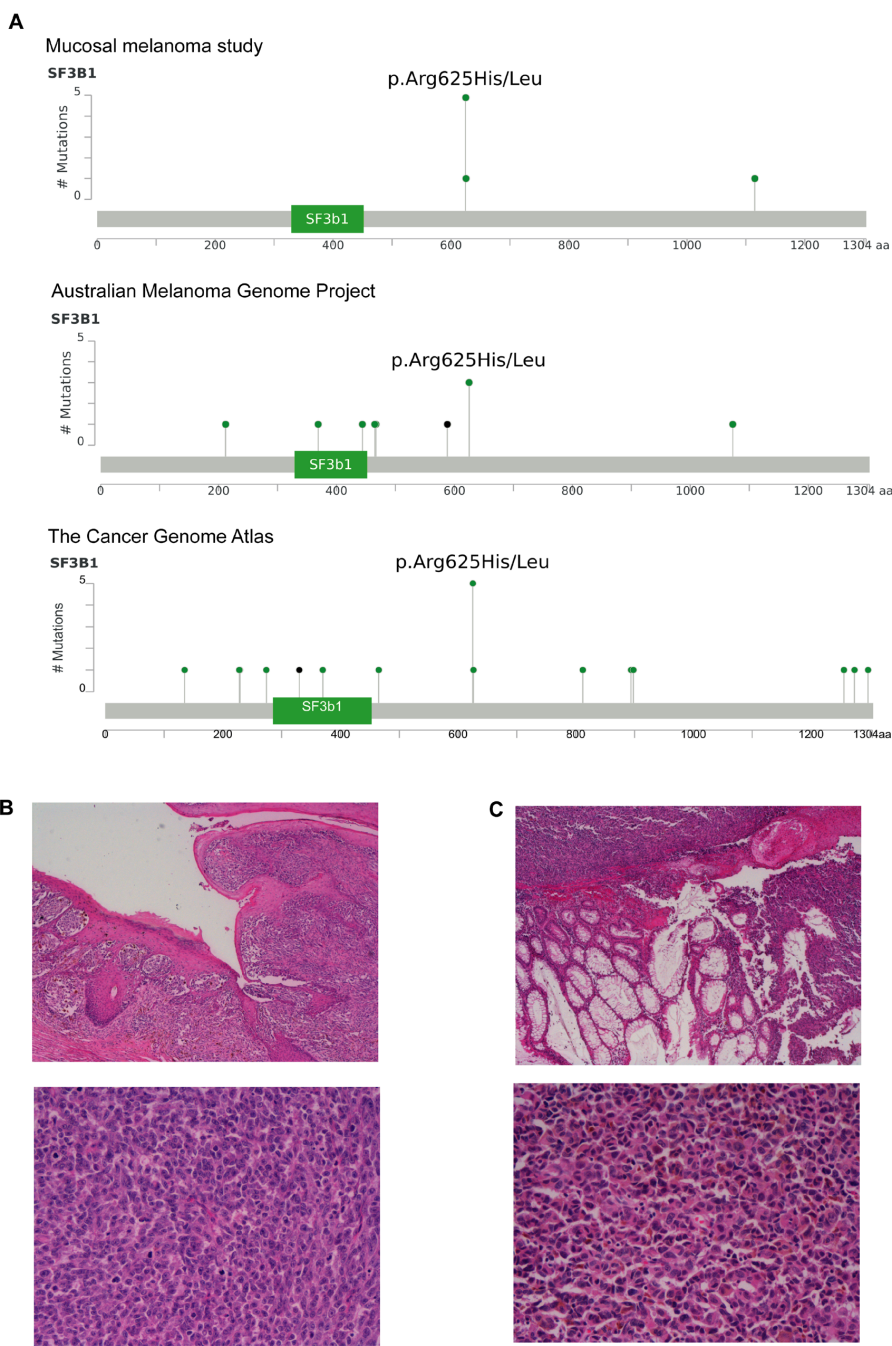
Recurrent hotspot SF3B1 R625 mutations and co-occurrence mutation events in mucosal melanoma (**A**) Lollipop plots showing amino acid changes (p.Arg625His/Leu) at codon 625 on SF3B1 protein structure in the present mucosal melanoma study, Australian Melanoma Genome Project and The Cancer Genome Atlas (SKCM-TCGA). The y-axis shows the number of mutations and the x-axis represent the amino acid residues of SF3B1 protein. Representative images of two cases (H & E x 4–upper panel, H & E x 20–lower panel) of mucosal melanoma with *SF3B1* mutation obtained from vulva (**B**) and (**C**) rectal sites.

We previously reported splicing factor *SF3B1* as a significantly mutated gene in mucosal melanoma using OncodriveFML (Hayward *et al.* 2017). Similar to The Cancer Genome Atlas cohort and our previous published work [15, 29], the recurrent *SF3B1* mutations occured at codon 625, comprising four p.R625H and one p.R625L alterations (Figure 2A). These recurrent *SF3B1* mutations were only identified in anorectal and vulva/vagina mucosal melanomas and not in melanoma samples from the conjunctiva, nasopharynx or palate (representative histological images in Figure 2B and 2C). The specific mutations found in each patient are described in Supplementary Table 2.

### Association of mutations with clinical and survival outcomes

**Figure 3:**
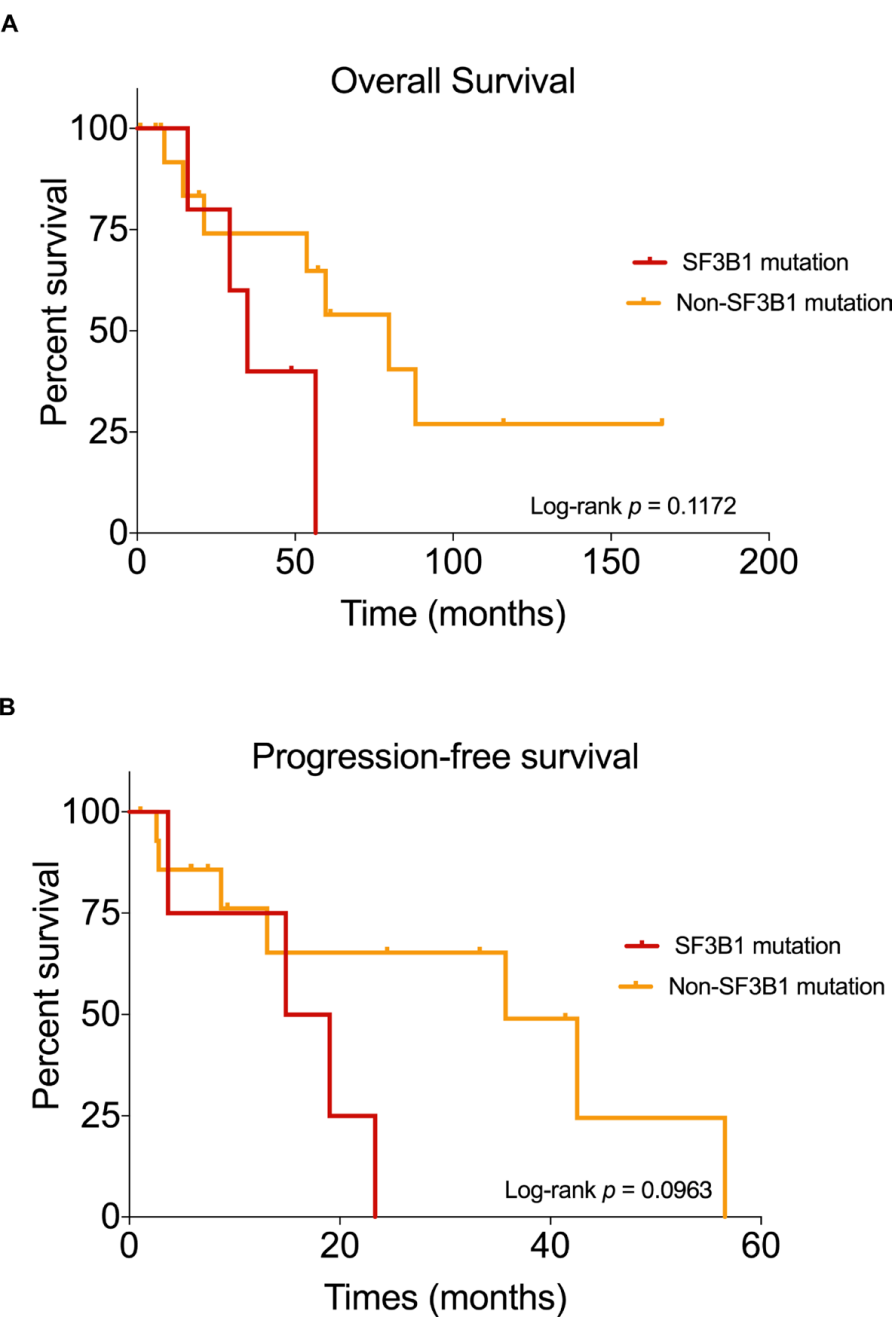
Kaplan–Meier curves showing the survival outcomes of mucosal melanoma patients Comparison of overall survival (**A**) and progression-free survival (**B**) in patients with mutated *SF3B1* and non-*SF3B1* mutation group.

The clinicopathological characteristics of tumors with *SF3B1* and non-*SF3B1* mutations are detailed and compared in Table 2. Representative histological images of *SF3B1* mutant tumors are presented in Figures 2B and 2C. Of all the patients with *SF3B1* mutations, two (33.3%) had T2 disease, one (16.7%) had T3 disease, and the remaining three (50%) had T4 disease. The depth of invasion of *SF3B1* mutant tumors ranged from 1.9 to 12 mm. Melanomas with *SF3B1* mutations had a similar mitotic rate when compared to non-*SF3B1* mutated cases (15.6±4.4 vs 15.5±2.54 mitoses/mm^2^) but were more often ulcerated (*SF3B1:* 6 out of 6 cases vs non-*SF3B1*: 10 of 16 cases with ulceration data), and were comprised of heterogeneous cell types. We tested whether *SF3B1* mutated and non-*SF3B1* cases had different prognosis. Median overall survival (OS) was 34.9 months in patients harboring *SF3B1* mutations compared to 79.7 months in patients with non-*SF3B1* mutations (HR: 2.44, 95% CI: 0.54 to 11, Log-rank test *P* = 0.117; Figure 3A). Median progression-free survival (PFS) was 16.9 months in the *SF3B1* mutant group and 35.7 months in non-*SF3B1* patients (HR: 0.474, 95% CI: 0.139 to 1.62, Log-rank test *P* = 0.0963; Figure 3B).

**Table 2:** Association of SF3B1 mutations with clinicopathological features

Characteristics	SF3B1 mutations identified (*n* = 6) *N* (%)^a^	Non-SF3B1 mutations identified (*n* = 21)^a^ *N* (%)^a^	*P*-value
Age (median, IQR)	52.5 years, 46.5–71.5 years	64 years, 54.5–79 years	0.309
Gender Female Male	4 (67%) 2 (33%)	18 (86%) 3 (14%)	0.303
Site Vulva/vagina Other sites (total) Anorectal Nasopharyngeal Conjunctiva Palate	3 (50%) 3 (50%) in total 2 (33%) 0 (0%) 1 (17%) 0 (0%)	12 (57%) 9 (43%) in total 3 (14%) 5 (24%) 0 (0%) 1 (5%)	>0.999
T classification 0–2 3–4 Data unavailable	2 (33%) 4 (67%) 0 (0%)	5 (24%) 13 (62%) 3 (14%)	>0.999
N classification N0 N+ Data unavailable	5 (83%) 0 (0%) 1 (17%)	12 (57%) 7 (33%) 2 (10%)	0.272
Mitotic rate (number of mitoses/mm2) < 10 ≥ 10 Data unavailable	1 (17%) 4 (67%) 1 (17%)	6 (29%) 10 (48%) 5 (24%)	0.624
Ulceration Absent Present Data unavailable	0 (0%) 6 (100%) 0 (0%)	6 (29%) 10 (48%) 5 (24%)	0.133
Tumor thickness (mm) <1 1–4 ≥ 4 Data unavailable	0 (0%) 3 (50%) 3 (50%) 0 (0%)	1 (5%) 6 (29%) 10 (48%) 4 (19%)	0.655^b^
Cell morphology Epithelioid Mixed Spindle Data unavailable	3 (50%) 2 (33%) 1 (17%) 0 (0%)	13 (62%) 1 (5%) 4 (19%) 3 (14%)	0.362^c^

^a^ Unless otherwise indicated.

^b^ Tissue with <1 mm is excluded from statistical testing.

^c^ Statistical testing was performed by comparing epithelioid and other cell morphology (mixed and spindle).

## DISCUSSION

In this study, 27 cases of mucosal melanoma were screened for mutations across 45 key oncogenes identified in our previous whole-genome sequencing study of major melanoma subtypes [15]. We found that *SF3B1* and *KIT* mutations predominantly occurred in melanomas originating in vulval/vaginal sites. Genes that were known to be mutated in cutaneous and uveal melanoma subtypes, including *BRAF*, *NRAS*, *NF1*, *GNAQ* and *GNA11*, were rarely mutated in our cohort of patients.

Mutations within exons 12–15 of *SF3B1*, encoding the C-terminal portion of the protein, have been described in 20% of uveal melanoma, 19% of CLL and 1.8% of breast cancers [20, 22, 30]. In our cohort of 27 mucosal melanoma patients, six (22%) carried a *SF3B1* mutation (in exons 14 and 23). Five patients harbored a mutation at exon 14, including p.R625H (*n* = 4) and p.R625L (*n* = 1), while one patient carried a p.C1123Y mutation within exon 23 in *SF3B1*. Of particular interest is the confirmation that the *SF3B1* hotspot mutant cases are apparently unique to mucosal melanomas of the lower body sites, hinting to divergent biology with those of upper body sites [24]. Furthermore, somatic mutations in *KIT* have been reported in 15–20% of mucosal melanomas, and are more commonly observed in anorectal and vulval/vaginal tumors (15–25%) [13, 31–33]. Our study supports these previous reports [13, 31–33], and detected *KIT* mutations in 11% of mucosal melanomas, particularly in the vulval and vaginal sites. KIT functions as a receptor tyrosine kinase, which transmits signals from the cell membrane into the cell [34, 35]. Once activated, KIT plays an important role in initiating the activation of MAPK/MEK and PI3K/AKT pathways that are critical in cancer development [36]. The aberrant MAPK/MEK and PI3K/AKT pathways impact a variety of cellular activities including cell proliferation and differentiation, which can result in neoplastic growth. Furthermore, besides SF3B1 and KIT mutations, other variants have been described in the context of mucosal melanoma, including amplifications of CCND1, MDM2 and KRAS ([15, 24]), however this was not assessed this cohort and could represent a source of additional driver eventsin this cohort.

Our findings are in line with the growing evidence that the recurrent hotspot mutation at codon 625 of *SF3B1* has functional impact in initiating aberrant 3’ splice site selection causing down-regulation of canonical protein expression to promote tumorigenesis [16–18, 20, 24]. The cancer-associated p.R625 mutation resulted in a conformational change in the U2 snRNP complex, such as p14 or U2AF, leading to the binding of alternative branchpoints for RNA splicing that processed these aberrant transcripts into aberrant proteins with altered functions [16, 17, 24, 25]. In melanoma, there has been significant interest in establishing whether the splicing inhibition contributes to cancer development and progression by specific pathologic splicing events. SF3B1 inhibitors have now been studied in patients with locally advanced or metastatic solid tumors. As an example, E7107 was tested in the phase I, open-label and single-arm clinical trial (Study E7107-A001-101; Trial registration ID: NCT00499499) for solid tumors, including colorectal, esophageal and pancreatic carcinomas [37, 38]. Pharmacodynamic analysis revealed that splicing inhibition in the peripheral blood mononuclear cells was achieved, and that it was dose dependant and reversible. Overall treatment was well tolerated, however, an unexpected toxicity of bilateral optic neuritis was detected in the patients, leading to the suspension of the clinical trial [37, 38]. Future design of clinical trials that include the spliceosome inhibitors should consider the risk of toxicity in conjunction with the on- and off-target effect of SF3B1 inhibition. Elucidating the impact of *SF3B1* mutation in mucosal melanomas may provide more understanding of its role in tumorigenesis, and facilitate the development of new drugs (i.e. *SF3B1* inhibitors) for mucosal melanomas with *SF3B1* mutations.

*SF3B1* mutations have different prognostic associations in different types of cancers [22, 24, 26, 30, 39, 40]. In uveal melanoma and myelodysplastic syndrome, *SF3B1* mutations are associated with a better prognosis, whereas in CLL, *SF3B1* mutations are correlated with a worse prognosis [17, 20, 40, 41]. In our study, *SF3B1*-mutated patients had a worse PFS and OS outcomes, however, our study of these rare tumors was underpowered to detect statistically significant differences. Larger studies are required to address this issue.

In summary, we discovered a *SF3B1* C1123Y mutation in a conjunctival mucosal melanoma, and a recurrent *SF3B1* R625 mutation, which predominantly occurred in female genital tract mucosal melanomas. We validated previous reports that the commonly known mutations in cutaneous melanoma, including *BRAF, NRAS, NF1, GNAQ* and *GNA11*, were rarely mutated in mucosal melanomas. This study provides additional insight into genetic alterations that occur in mucosal melanomas. Collectively, these findings provide a better understanding of the oncogenic drivers of tumor development and may the identification of effective systemic therapies for these rare melanomas.

## MATERIALS AND METHODS

### Patients and sample selection

This study was approved by the New South Wales Department of Health Human Research Ethics Committee (Protocol no. X15-0454). All human research procedures were performed according to the National Health and Medical Research Council of Australia guidelines. Samples were acquired with patients’ informed consent from the Melanoma Institute Australia Biospecimen bank and Royal Prince Alfred Hospital. Mucosal melanomas were defined as occurring in the mucosa membranes of the oral, conjunctiva, respiratory, gastrointestinal and urogenital tracts. The Melanoma Institute Australia’s research database was searched for primary melanomas originating in the aforementioned sites, and all available archival formalin-fixed biopsies were collected from these patients. The H&E slides of the primary melanoma of all cases were carefully reviewed independently by melanoma pathologists (R.V.R, P.M.F, and R.A.S) to ensure they were from a primary mucosal site. Any melanoma that had arisen at the junction of mucosa and cutaneous skin was excluded.

### DNA isolation

Formalin-fixed, paraffin-embedded tumor tissue was prepared in 10 μm sections and deparaffinized through xylene and ethanol according to standard procedures. After air-drying, the tumor tissue was manually macro-dissected, as previously reported [42]. Genomic DNA was isolated using AllPrep DNA/RNA kits (Qiagen) according to the manufacturer’s instructions. Samples were quantified using the Qubit dsDNA high sensitivity assay (Life Technologies).

### Amplicon library construction and Illumina sequencing

Amplicon dual-strand (DS) library preparation was performed using the TruSeq Custom Amplion Low Input Library Prep Kit according to the manufacturer’s protocol. Briefly, the amount of DNA was quantitated and diluted to a final concentration of 10−25 ng/μl. The DNA samples were hybridized to the custom amplicons across 45 target genes (Supplementary Table 3) using Illumina’s recommended HYB temperature gradient program from 90°C to 40°C. Subsequently, unbound oligos were washed using Stringent Wash Buffer 1 and 60% ethanol, and was followed by extension and ligation of targeted regions of interest. For individual libraries, the extension-ligation products were ligated to i7 and i5 adapters containing an unique indexed sequence, and followed by library amplification. The amplified paired-end DS libraries were pooled and each strand was sequenced independently using the Illumina MiniSeq™ instrument (Illumina).

### Amplicon sequence processing and somatic variant analysis

Sequence data were aligned to the UCSC hg19 assembly using BWA 0.7.13 [43], and SAMtools 1.3 [44] was used to convert aligned sequence read format to binary file (BAM) for each respective sample. These BAMs were marked for duplicate reads using Picard 2.1.1 [45]. Somatic variants were detected using Illumina Amplicon DS Somatic Variant Caller. Briefly, the algorithm analyzed each pool separately, and computed the variant scores assuming that the non-reference calls should follow a Poisson distribution: *P* = 1–CDF(K -1, λ), where P is the probability that no variant is present given K or more observations, and CDF represents Poisson cumulative distribution function. The probability, P, is subsequently converted to a *Q*-score and only calls with above Q20 were included for further processing. Variants that were marked as passing quality control contained the following criteria: (i) each variant present in both pools, (ii) a cumulative depth of 1000, and (iii) at least 3% or greater variant frequency. Variants were annotated using human genome build UCSC hg19 with ANNOVAR [46]. Variants were further parsed to OncodriveMut in Cancer Genome Interpreter to identify biological and clinical relevance somatic genes [47]. The mutation results and somatic interactions were further analyzed and visualized using R package maftools [48].

### Statistical analysis

The association of mutation status with available clinicopathologic characteristics was performed using Mann–Whitney *U* test and Fischer exact tests for continuous and categorical variables as appropriate. The Kaplan–Meier survival curves were compared according to the type of mutation group using the log-rank test. All statistical analyses were performed using GraphPad Prism 7.0.

## SUPPLEMENTARY MATERIALS TABLES







## Abbreviations

AKT: Protein kinase B
ARID2: AT-Rich Interaction Domain 2
BRAF: Serine/threonine-protein kinase B-raf
C: Cysteine
CI: Confidence interval
CLL: Chronic lymphocytic leukemia
CTNNB1: Catenin Beta 1
DICER1: Ribonuclease III
DNA: Deoxyribonucleic acid
DS: Dual strand
E: Glutamic acid
GNA11: G Protein Subunit Alpha 11
GNAQ: G Protein Subunit Alpha Q
H: Histidine
hg19: human genome reference version 19
HR: Hazard ratio
K: Lysine
KIT: Proto-Oncogene Receptor Tyrosine Kinase
L: Leucine
MAP2K1/MEK/MAPK: Mitogen-Activated Protein Kinase Kinase 1
NF1: Neurofibromatosis 1
NRAS: neuroblastoma RAS viral oncogene homolog
OS: Overall survival
P: Proline
PFS: Progression-free survival
PI3K: Phosphoinositide 3-kinase
PTEN: Phosphatase And Tensin Homolog
R: Arginine
RNA: Ribonucleic acid
SETD2: SET(Su(var)3-9, Enhancer-of-zeste and Trithorax) Domain Containing 2
SF3B1: Splicing factor 3B subunit 1
T: Threonine
TP53: Tumor protein p53
V: Valine
Y: Tyrosine
