# Supplementary material for: Recurrent hotspot SF3B1 mutations at codon 625 in vulvovaginal mucosal melanoma identified in a study of 27 Australian mucosal melanomas
Source: Oncotarget. 2019 Jan 29;10(9):930–41. doi: 10.18632/oncotarget.26584 (PMC6398173; doi:10.18632/oncotarget.26584)
Supplement: Supplementary file 1 [file oncotarget-10-930-s001.pdf]

Recurrent hotspot SF3B1 mutations at codon 625 in vulvovaginal mucosal melanoma identified in a study of 27 Australian mucosal melanomas

SUPPLEMENTARY MATERIALS

Supplementary Table 1: Clinical and pathological parameters of mucosal melanoma patients. See Suppleemntary_Table_1

Supplementary Table 2: Genetic alterations identified in mucosal melanoma

Patient ID	Gene	Exon:Nucleotide change:Amino acid change	Oncogenic classification (oncodriveMUT)
Patient 10	SETD2	exon4:c.G4487A:p.R1496Q	predicted driver: tier 1
Patient 18	TP53	exon6:c.A578T:p.H193L	predicted driver: tier 1
Patient 5	KIT	exon11:c.T1727C:p.L576P	known in: THYM;CM
Patient 11	KIT	exon11:c.T1727C:p.L576P	known in: THYM;CM
Patient 17	KIT	exon14:c.C2009T:p.T670I	known in: GIST
Patient 1	SF3B1	exon14:c.G1874T:p.R625L	predicted driver: tier 1
Patient 2	SF3B1	exon14:c.G1874A:p.R625H	known in: CANCER
Patient 9	SF3B1	exon14:c.G1874A:p.R625H	known in: CANCER
Patient 10	SF3B1	exon23:c.G3368A:p.C1123Y	predicted driver: tier 1
Patient 12	SF3B1	exon14:c.G1874A:p.R625H	known in: CANCER
Patient 22	SF3B1	exon14:c.G1874A:p.R625H	known in: CANCER
Patient 23	ARID2	exon15:c.C2458T:p.Q820X	predicted driver: tier 1
Patient 1	BRAF	exon12:c.A1447G:p.K483E	predicted driver: tier 2
Patient 23	BRAF	exon15:c.G1780A:p.D594N	known in: CM
Patient 8	CTNNB1	exon11:c.G1687A:p.G563R	predicted driver: tier 1
Patient 22	NF1	exon28:c.T3824C:p.F1275S	predicted driver: tier 2
Patient 21	NRAS	exon3:c.A182T:p.Q61L	known in: NSCLC; CM
Patient 25	NRAS	exon3:c.C181A:p.Q61K	known in: NSCLC;AML;THCA;COREAD;CM
Patient 19	DICER1	exon22:c.G4702A:p.G1568S	predicted driver: tier 1
Patient 1	MAP2K1	exon11:c.G1168A:p.A390T	known in: COREAD
Patient 26	PTEN	exon8:c.863dupA:p.V290Sfs*8	predicted driver: tier 1

Abbreviations: AML – Acute myeloid leukaemia, COREAD – Colorectal adenocarcinoma, CM – Cutaneous melanoma, GIST – Gastrointestinal stromal tumors, NSCLC – Non-small cell lung cancer, THCA – Thyroid carcinoma, THYM – Thymoma.

Supplementary Table 3: A custom amplicon panels designed for targeted DNA sequencing. See Suppleemntary_Table_3
