# Supplementary material for: Recurrent hotspot SF3B1 mutations at codon 625 in vulvovaginal mucosal melanoma identified in a study of 27 Australian mucosal melanomas
Source: Oncotarget. 2019 Jan 29;10(9):930–41. doi: 10.18632/oncotarget.26584 (PMC6398173; doi:10.18632/oncotarget.26584)
Supplement: Supplementary file 2 [file oncotarget-10-930-s002.docx]

**Supplementary Table 1. Clinical and pathological parameters of mucosal melanoma patients.**

| **Patient ID** | **Primary Site** | **Progression Free Survival (months)** | **Overall survival (months)** | **Age at diagnosis** | **Gender** | **Ulceration (Absent / Present)** | **Mitotic rate (number of mitoses/mm2)** | **Depth of invasion (mm)** | **Cell type** | **Tumor stage (AJCC 8th Ed.*)** |
| --- | --- | --- | --- | --- | --- | --- | --- | --- | --- | --- |
| Patient 1 | Vulva | 33.6 | 48.8 | 54 | Female | Present | 4 | 1.9 | Mixed | IIA (T2b, N0, M0) |
| Patient 2 | Rectum | 19.0 | 34.9 | 66 | Female | Present | 12 | 4.2 | Epithelioid | IIC (T4b, N0, M0) |
| Patient 3 | Rectum | 2.8 | 8.6 | 68 | Female | Present | 20 | 42 | Epithelioid | IIC (T4b, N0, M0) |
| Patient 4 | Vulva | NA | NA | 61 | Female | Present | 3 | 4.5 | Epithelioid | IIIC (T4b, N1a, M0) |
| Patient 5 | Vulva | 42.5 | 88.1 | 87 | Female | Absent | 4 | 1.45 | Epithelioid | IB (T2a, N0, M0) |
| Patient 6 | P/nasal Sinus | 35.7 | 79.7 | 64 | Female | Present | NA | NA | Epithelioid | NA |
| Patient 7 | Vulva | 7.5 | 7.5 | 56 | Female | Present | 15 | 9 | Epithelioid | IIIC (T4b, N1, M0) |
| Patient 8 | Palate | 13.1 | 21.2 | 82 | Male | Present | 5 | 1.8 | Spindle | IIA (T2b, N0, M0) |
| Patient 9 | Vulva | 14.9 | 56.5 | 51 | Female | Present | 12 | 2 | Mixed | IIA (T2b, N0, M0) |
| Patient 10 | Conjunctiva | 3.7 | 16.0 | 42 | Male | Present | 30 | 3.5 | Epithelioid | IIB (T3b, N0, M0) |
| Patient 11 | Vulva | NA | NA | 90 | Female | Present | 16 | 8.6 | Spindle | IIIC (T4b, N2b, M0) |
| Patient 12 | Vulva | NA | NA | 88 | Female | Present | 20 | 10 | Spindle | IIC (T4b, NX, MX) |
| Patient 13 | Vulva | 5.9 | 5.9 | 29 | Female | Present | 20 | 7.5 | Epithelioid | IIIC (T4b, N2a, M0) |
| Patient 14 | Vagina | 24.5 | 116.0 | 60 | Female | Absent | 8 | 7 | Mixed | IIC (T4a, N0, M0) |
| Patient 15 | Vulva | 5.9 | 19.5 | 75 | Female | Absent | 19 | 8 | Epithelioid | IIIC (T4a, N1, M0) |
| Patient 16 | Nasal | NA | NA | 73 | Female | NA | NA | NA | NA | NA |
| Patient 17 | Anus | 8.7 | 14.5 | 32 | Female | Present | 16 | 2.7 | Epithelioid | IV (T3b, N2a, M1b) |
| Patient 18 | Vulva | 1.1 | 1.1 | 80 | Female | Absent | 4 | 1.6 | Epithelioid | IIA (T3a, N0, M0) |
| Patient 19 | Anus | 2.6 | 53.7 | 60 | Female | Present | 25 | 8 | NA | IIC (T4b, N0, M0) |
| Patient 20 | Vulva | NA | NA | 65 | Female | Absent | 35 | 5.4 | Epithelioid | IIB (T4a, N0, M0) |
| Patient 21 | Nasal Cavity | 9.3 | 166.2 | 49 | Female | NA | NA | 0.3 | NA | IA (T1a, N0, M0) |
| Patient 22 | Rectum | 23.3 | 29.3 | 48 | Male | Present | NA | 12 | Epithelioid | IIC (T4b, N0, M0) |
| Patient 23 | Vulva | NA | NA | 90 | Female | Absent | 8 | 4.1 | Epithelioid | IIB (T4a, N0, M0) |
| Patient 24 | Nasal Cavity | 33.3 | 57.3 | 58 | Male | NA | NA | NA | Spindle | IIIB (T0, N1b, M0) |
| Patient 25 | Nasal Cavity | 41.4 | 61.2 | 53 | Male | Present | 15 | 1.1 | Epithelioid | IIA (T2b, N0, M0) |
| Patient 26 | Vaginal | NA | NA | 78 | Female | NA | NA | NA | Epithelioid | NA |
| Patient 27 | Vagina | 56.6 | 59.7 | 45 | Female | NA | 35 | 4 | Spindle | IIB (T4a, N0, M0) |

'NA' indicates Not Available

*According to cutenous staging system. Gershenwald JE, Scolyer RA, et al. Melanoma staging: Evidence‐based changes in the American Joint Committee on Cancer eighth edition cancer staging manual. CA: a cancer journal for clinicians. 2017 Nov;67(6):472-92.
