# Supplementary material for: Recurrent hotspot SF3B1 mutations at codon 625 in vulvovaginal mucosal melanoma identified in a study of 27 Australian mucosal melanomas
Source: Oncotarget. 2019 Jan 29;10(9):930–41. doi: 10.18632/oncotarget.26584 (PMC6398173; doi:10.18632/oncotarget.26584)
Supplement: Supplementary file 3 [file oncotarget-10-930-s003.docx]

Supplementary Table 3. A custom amplicon panels designed for targeted DNA sequencing.

| **Target** | **Chromosome** | **Start Coordinate** | **Stop Coordinate** | **Length** |
| --- | --- | --- | --- | --- |
| ZFX | X | 24190860 | 24190917 | 58 |
| ZFX | X | 24226335 | 24226487 | 153 |
| ZFX | X | 24197300 | 24197887 | 588 |
| ZFX | X | 24225829 | 24225972 | 144 |
| CASP8 | 2 | 202139612 | 202139676 | 65 |
| CASP8 | 2 | 202151182 | 202151314 | 133 |
| CASP8 | 2 | 202134233 | 202134328 | 96 |
| CASP8 | 2 | 202149539 | 202150040 | 502 |
| CASP8 | 2 | 202136239 | 202136344 | 106 |
| CASP8 | 2 | 202122955 | 202123105 | 151 |
| CWH43 | 4 | 48988447 | 48988489 | 43 |
| CWH43 | 4 | 49019266 | 49019345 | 80 |
| CWH43 | 4 | 49030646 | 49030751 | 106 |
| CWH43 | 4 | 49009218 | 49009343 | 126 |
| CWH43 | 4 | 49032842 | 49032977 | 136 |
| CWH43 | 4 | 49000477 | 49000565 | 89 |
| CWH43 | 4 | 49063829 | 49063904 | 76 |
| CWH43 | 4 | 49034583 | 49034732 | 150 |
| CWH43 | 4 | 48993953 | 48994107 | 155 |
| CWH43 | 4 | 49052711 | 49052866 | 156 |
| CWH43 | 4 | 48996636 | 48996837 | 202 |
| CWH43 | 4 | 49046803 | 49046864 | 62 |
| CWH43 | 4 | 48993471 | 48993591 | 121 |
| CWH43 | 4 | 48990494 | 48990685 | 192 |
| CWH43 | 4 | 49005752 | 49006009 | 258 |
| CWH43 | 4 | 49040053 | 49040197 | 145 |
| SH3BP1 | 22 | 38037133 | 38037175 | 43 |
| SH3BP1 | 22 | 38037381 | 38037485 | 105 |
| SH3BP1 | 22 | 38044326 | 38044442 | 117 |
| SH3BP1 | 22 | 38041372 | 38041517 | 146 |
| SH3BP1 | 22 | 38046159 | 38046256 | 98 |
| SH3BP1 | 22 | 38035795 | 38035853 | 59 |
| SH3BP1 | 22 | 38046549 | 38046732 | 184 |
| SH3BP1 | 22 | 38039651 | 38039795 | 145 |
| SH3BP1 | 22 | 38042825 | 38042936 | 112 |
| SH3BP1 | 22 | 38049786 | 38049880 | 95 |
| SH3BP1 | 22 | 38038546 | 38038622 | 77 |
| SH3BP1 | 22 | 38051279 | 38051688 | 410 |
| EPHB3 | 3 | 184296202 | 184296254 | 53 |
| EPHB3 | 3 | 184280024 | 184280141 | 118 |
| EPHB3 | 3 | 184293618 | 184293773 | 156 |
| EPHB3 | 3 | 184289100 | 184289190 | 91 |
| EPHB3 | 3 | 184290290 | 184290985 | 696 |
| MAP2K2 | 19 | 4090599 | 4090706 | 108 |
| MAP2K2 | 19 | 4123781 | 4123872 | 92 |

| MAP2K2 | 19 | 4102374 | 4102451 | 78 |
| --- | --- | --- | --- | --- |
| MAP2K2 | 19 | 4099199 | 4099412 | 214 |
| MAP2K2 | 19 | 4095386 | 4095447 | 62 |
| MAP2K2 | 19 | 4097277 | 4097341 | 65 |
| MAP2K2 | 19 | 4117417 | 4117627 | 211 |
| MAP2K2 | 19 | 4094451 | 4094496 | 46 |
| MAP2K2 | 19 | 4110507 | 4110653 | 147 |
| GNA11 | 19 | 3120987 | 3121174 | 188 |
| GNA11 | 19 | 3114942 | 3115070 | 129 |
| GNA11 | 19 | 3113328 | 3113482 | 155 |
| GNA11 | 19 | 3110147 | 3110331 | 185 |
| GNA11 | 19 | 3094600 | 3094850 | 251 |
| KIT | 4 | 55564450 | 55564731 | 282 |
| KIT | 4 | 55599236 | 55599358 | 123 |
| KIT | 4 | 55524182 | 55524248 | 67 |
| KIT | 4 | 55597494 | 55597585 | 92 |
| KIT | 4 | 55569890 | 55570058 | 169 |
| KIT | 4 | 55561650 | 55561955 | 306 |
| KIT | 4 | 55603341 | 55603446 | 106 |
| KIT | 4 | 55589750 | 55589864 | 115 |
| KIT | 4 | 55575590 | 55575705 | 116 |
| KIT | 4 | 55604595 | 55604720 | 126 |
| KIT | 4 | 55573264 | 55573453 | 190 |
| KIT | 4 | 55565796 | 55565932 | 137 |
| KIT | 4 | 55595501 | 55595651 | 151 |
| KIT | 4 | 55598037 | 55598164 | 128 |
| SF3B1 | 2 | 198265439 | 198265660 | 222 |
| SF3B1 | 2 | 198257030 | 198257185 | 156 |
| SF3B1 | 2 | 198257680 | 198257960 | 281 |
| SF3B1 | 2 | 198262709 | 198262840 | 132 |
| SF3B1 | 2 | 198281465 | 198281635 | 171 |
| SF3B1 | 2 | 198283233 | 198283312 | 80 |
| SF3B1 | 2 | 198288532 | 198288698 | 167 |
| SF3B1 | 2 | 198274494 | 198274731 | 238 |
| SF3B1 | 2 | 198266124 | 198266249 | 126 |
| SF3B1 | 2 | 198299696 | 198299723 | 28 |
| SF3B1 | 2 | 198283659 | 198283675 | 17 |
| SF3B1 | 2 | 198268309 | 198268488 | 180 |
| SF3B1 | 2 | 198285753 | 198285857 | 105 |
| SF3B1 | 2 | 198285152 | 198285266 | 115 |
| SF3B1 | 2 | 198263185 | 198263305 | 121 |
| SF3B1 | 2 | 198260780 | 198261052 | 273 |
| SF3B1 | 2 | 198273093 | 198273305 | 213 |
| SF3B1 | 2 | 198272722 | 198272843 | 122 |
| NNT | 5 | 43655942 | 43656175 | 234 |
| NNT | 5 | 43644713 | 43644904 | 192 |
| NNT | 5 | 43700221 | 43700339 | 119 |
| NNT | 5 | 43613010 | 43613239 | 230 |

| NNT | 5 | 43677827 | 43677908 | 82 |
| --- | --- | --- | --- | --- |
| NNT | 5 | 43702723 | 43702838 | 116 |
| NNT | 5 | 43659273 | 43659452 | 180 |
| NNT | 5 | 43645459 | 43645612 | 154 |
| NNT | 5 | 43704357 | 43704503 | 147 |
| NNT | 5 | 43609298 | 43609448 | 151 |
| NNT | 5 | 43650579 | 43650689 | 111 |
| NNT | 5 | 43644294 | 43644427 | 134 |
| NNT | 5 | 43656755 | 43656915 | 161 |
| NNT | 5 | 43628302 | 43628489 | 188 |
| NNT | 5 | 43619134 | 43619221 | 88 |
| NNT | 5 | 43651841 | 43651986 | 146 |
| NNT | 5 | 43615950 | 43616167 | 218 |
| NNT | 5 | 43624100 | 43624250 | 151 |
| NNT | 5 | 43675613 | 43675772 | 160 |
| NNT | 5 | 43649249 | 43649410 | 162 |
| NNT | 5 | 43653120 | 43653315 | 196 |
| RASA2 | 3 | 141235170 | 141235273 | 104 |
| RASA2 | 3 | 141248550 | 141248644 | 95 |
| RASA2 | 3 | 141291451 | 141291565 | 115 |
| RASA2 | 3 | 141299942 | 141300019 | 78 |
| RASA2 | 3 | 141328262 | 141328365 | 104 |
| RASA2 | 3 | 141290248 | 141290396 | 149 |
| RASA2 | 3 | 141295842 | 141295948 | 107 |
| RASA2 | 3 | 141289754 | 141289910 | 157 |
| RASA2 | 3 | 141299210 | 141299293 | 84 |
| RASA2 | 3 | 141231005 | 141231122 | 118 |
| RASA2 | 3 | 141205926 | 141206058 | 133 |
| RASA2 | 3 | 141328719 | 141328908 | 190 |
| RASA2 | 3 | 141274682 | 141274754 | 73 |
| RASA2 | 3 | 141277728 | 141277804 | 77 |
| RASA2 | 3 | 141331125 | 141331152 | 28 |
| RASA2 | 3 | 141259375 | 141259451 | 77 |
| RASA2 | 3 | 141305488 | 141305594 | 107 |
| RASA2 | 3 | 141304867 | 141304940 | 74 |
| RASA2 | 3 | 141327331 | 141327539 | 209 |
| RASA2 | 3 | 141291989 | 141292063 | 75 |
| RASA2 | 3 | 141278736 | 141278837 | 102 |
| RASA2 | 3 | 141292786 | 141292909 | 124 |
| RASA2 | 3 | 141326520 | 141326602 | 83 |
| RASA2 | 3 | 141272699 | 141272782 | 84 |
| CCND1 | 11 | 69462762 | 69462910 | 149 |
| CCND1 | 11 | 69456082 | 69456279 | 198 |
| CCND1 | 11 | 69465886 | 69466047 | 162 |
| CCND1 | 11 | 69457799 | 69458014 | 216 |
| CCND1 | 11 | 69458600 | 69458759 | 160 |
| RAC1 | 7 | 6439757 | 6439819 | 63 |
| RAC1 | 7 | 6414367 | 6414401 | 35 |

| RAC1 | 7 | 6431555 | 6431672 | 118 |
| --- | --- | --- | --- | --- |
| RAC1 | 7 | 6441499 | 6441658 | 160 |
| RAC1 | 7 | 6441947 | 6442074 | 128 |
| RAC1 | 7 | 6438293 | 6438349 | 57 |
| RAC1 | 7 | 6426843 | 6426914 | 72 |
| MAP2K1 | 15 | 66782820 | 66783000 | 181 |
| MAP2K1 | 15 | 66729084 | 66729230 | 147 |
| MAP2K1 | 15 | 66782056 | 66782101 | 46 |
| MAP2K1 | 15 | 66777328 | 66777529 | 202 |
| MAP2K1 | 15 | 66774093 | 66774217 | 125 |
| MAP2K1 | 15 | 66736994 | 66737045 | 52 |
| MAP2K1 | 15 | 66779566 | 66779630 | 65 |
| MAP2K1 | 15 | 66735618 | 66735695 | 78 |
| MAP2K1 | 15 | 66781553 | 66781614 | 62 |
| MAP2K1 | 15 | 66679686 | 66679765 | 80 |
| MAP2K1 | 15 | 66727365 | 66727575 | 211 |
| GNAQ | 9 | 80343430 | 80343583 | 154 |
| GNAQ | 9 | 80412436 | 80412564 | 129 |
| GNAQ | 9 | 80430532 | 80430686 | 155 |
| GNAQ | 9 | 80409379 | 80409508 | 130 |
| GNAQ | 9 | 80537077 | 80537261 | 185 |
| GNAQ | 9 | 80336242 | 80336429 | 188 |
| GNAQ | 9 | 80646016 | 80646151 | 136 |
| RB1 | 13 | 48953730 | 48953780 | 51 |
| DICER1 | 14 | 95574250 | 95574430 | 181 |
| DICER1 | 14 | 95573900 | 95574050 | 151 |
| SETD2 | 3 | 47125210 | 47125820 | 611 |
| SETD2 | 3 | 47155335 | 47155510 | 176 |
| SETD2 | 3 | 47161950 | 47163410 | 1461 |
| DDX3X | X | 41204400 | 41204800 | 401 |
| TP53 | 17 | 7576790 | 7577160 | 371 |
| NF1 | 17 | 29679275 | 29679432 | 158 |
| NF1 | 17 | 29541469 | 29541603 | 135 |
| NF1 | 17 | 29528055 | 29528177 | 123 |
| NF1 | 17 | 29527440 | 29527613 | 174 |
| NF1 | 17 | 29652800 | 29653270 | 471 |
| NF1 | 17 | 29508728 | 29508803 | 76 |
| NF1 | 17 | 29496909 | 29497015 | 107 |
| NF1 | 17 | 29654517 | 29654857 | 341 |
| NF1 | 17 | 29665722 | 29665823 | 102 |
| NF1 | 17 | 29550462 | 29550585 | 124 |
| NF1 | 17 | 29676138 | 29676269 | 132 |
| NF1 | 17 | 29554236 | 29554309 | 74 |
| NF1 | 17 | 29670027 | 29670153 | 127 |
| NF1 | 17 | 29528429 | 29528503 | 75 |
| NF1 | 17 | 29576002 | 29576137 | 136 |
| NF1 | 17 | 29554541 | 29554624 | 84 |
| NF1 | 17 | 29592247 | 29592357 | 111 |

| NF1 | 17 | 29585362 | 29585520 | 159 |
| --- | --- | --- | --- | --- |
| NF1 | 17 | 29579956 | 29580018 | 63 |
| NF1 | 17 | 29587387 | 29587533 | 147 |
| NF1 | 17 | 29685987 | 29686033 | 47 |
| NF1 | 17 | 29683478 | 29683600 | 123 |
| NF1 | 17 | 29552113 | 29552268 | 156 |
| NF1 | 17 | 29509526 | 29509683 | 158 |
| NF1 | 17 | 29546023 | 29546136 | 114 |
| NF1 | 17 | 29508440 | 29508507 | 68 |
| NF1 | 17 | 29557278 | 29557400 | 123 |
| NF1 | 17 | 29533258 | 29533389 | 132 |
| NF1 | 17 | 29685498 | 29685640 | 143 |
| NF1 | 17 | 29701031 | 29701170 | 140 |
| NF1 | 17 | 29422328 | 29422387 | 60 |
| NF1 | 17 | 29667523 | 29667663 | 141 |
| NF1 | 17 | 29556043 | 29556483 | 441 |
| NF1 | 17 | 29559091 | 29559207 | 117 |
| NF1 | 17 | 29586000 | 29586147 | 148 |
| NF1 | 17 | 29588729 | 29588875 | 147 |
| NF1 | 17 | 29483001 | 29483144 | 144 |
| NF1 | 17 | 29687505 | 29687721 | 217 |
| NF1 | 17 | 29486028 | 29486111 | 84 |
| NF1 | 17 | 29556853 | 29556992 | 140 |
| NF1 | 17 | 29661856 | 29662049 | 194 |
| NF1 | 17 | 29553453 | 29553702 | 250 |
| NF1 | 17 | 29557860 | 29557943 | 84 |
| NF1 | 17 | 29657314 | 29657516 | 203 |
| NF1 | 17 | 29664386 | 29664600 | 215 |
| NF1 | 17 | 29677201 | 29677336 | 136 |
| ARID2 | 12 | 46298717 | 46298858 | 142 |
| ARID2 | 12 | 46211453 | 46211671 | 219 |
| ARID2 | 12 | 46215203 | 46215270 | 68 |
| ARID2 | 12 | 46254584 | 46254732 | 149 |
| ARID2 | 12 | 46243363 | 46243559 | 197 |
| ARID2 | 12 | 46125000 | 46125097 | 98 |
| ARID2 | 12 | 46243790 | 46246700 | 2911 |
| ARID2 | 12 | 46233112 | 46233279 | 168 |
| ARID2 | 12 | 46205201 | 46205334 | 134 |
| ARID2 | 12 | 46242619 | 46242753 | 135 |
| ARID2 | 12 | 46240639 | 46240720 | 82 |
| CDKN2A | 9 | 21994138 | 21994330 | 193 |
| CDKN2A | 9 | 21968727 | 21968770 | 44 |
| CDKN2A | 9 | 21968231 | 21968241 | 11 |
| NRAS | 1 | 115251159 | 115251275 | 117 |
| NRAS | 1 | 115252190 | 115252349 | 160 |
| NRAS | 1 | 115256421 | 115256599 | 179 |
| NRAS | 1 | 115258671 | 115258781 | 111 |
| BRAF | 7 | 140508692 | 140508795 | 104 |

| BRAF | 7 | 140507760 | 140507862 | 103 |
| --- | --- | --- | --- | --- |
| BRAF | 7 | 140453987 | 140454033 | 47 |
| BRAF | 7 | 140476712 | 140476888 | 177 |
| BRAF | 7 | 140449087 | 140449218 | 132 |
| BRAF | 7 | 140481376 | 140481493 | 118 |
| BRAF | 7 | 140534409 | 140534672 | 264 |
| BRAF | 7 | 140453075 | 140453193 | 119 |
| BRAF | 7 | 140500162 | 140500281 | 120 |
| BRAF | 7 | 140501212 | 140501360 | 149 |
| BRAF | 7 | 140482821 | 140482957 | 137 |
| BRAF | 7 | 140549911 | 140550012 | 102 |
| BRAF | 7 | 140487348 | 140487384 | 37 |
| BRAF | 7 | 140494108 | 140494267 | 160 |
| BRAF | 7 | 140624366 | 140624503 | 138 |
| BRAF | 7 | 140434400 | 140434570 | 171 |
| BRAF | 7 | 140439612 | 140439746 | 135 |
| BRAF | 7 | 140477791 | 140477875 | 85 |
| DICER1 | 14 | 95582004 | 95582158 | 155 |
| DICER1 | 14 | 95590533 | 95591005 | 473 |
| DICER1 | 14 | 95599652 | 95599795 | 144 |
| DICER1 | 14 | 95562120 | 95563060 | 941 |
| DICER1 | 14 | 95572015 | 95572120 | 106 |
| DICER1 | 14 | 95582790 | 95583032 | 243 |
| DICER1 | 14 | 95566117 | 95566272 | 156 |
| DICER1 | 14 | 95560200 | 95560500 | 301 |
| DICER1 | 14 | 95596395 | 95596529 | 135 |
| DICER1 | 14 | 95592917 | 95593085 | 169 |
| DICER1 | 14 | 95595809 | 95595969 | 161 |
| DICER1 | 14 | 95572378 | 95572560 | 183 |
| DICER1 | 14 | 95579429 | 95579561 | 133 |
| DICER1 | 14 | 95569630 | 95570475 | 846 |
| DICER1 | 14 | 95574661 | 95574840 | 180 |
| DICER1 | 14 | 95578509 | 95578584 | 76 |
| DICER1 | 14 | 95583959 | 95584091 | 133 |
| DICER1 | 14 | 95597846 | 95597976 | 131 |
| DICER1 | 14 | 95598852 | 95599014 | 163 |
| DICER1 | 14 | 95571408 | 95571583 | 176 |
| DICER1 | 14 | 95577654 | 95577793 | 140 |
| HDAC9 | 7 | 18806729 | 18806778 | 50 |
| HDAC9 | 7 | 18498465 | 18498524 | 60 |
| HDAC9 | 7 | 18633531 | 18633652 | 122 |
| HDAC9 | 7 | 18875090 | 18875209 | 120 |
| HDAC9 | 7 | 18668973 | 18669104 | 132 |
| HDAC9 | 7 | 18688089 | 18688306 | 218 |
| HDAC9 | 7 | 19035646 | 19035682 | 37 |
| HDAC9 | 7 | 18832968 | 18833075 | 108 |
| HDAC9 | 7 | 18788628 | 18788761 | 134 |
| HDAC9 | 7 | 18674250 | 18674365 | 116 |

| HDAC9 | 7 | 18975432 | 18975565 | 134 |
| --- | --- | --- | --- | --- |
| HDAC9 | 7 | 18684294 | 18684416 | 123 |
| HDAC9 | 7 | 19015429 | 19015576 | 148 |
| HDAC9 | 7 | 18801780 | 18801900 | 121 |
| HDAC9 | 7 | 18875523 | 18875620 | 98 |
| HDAC9 | 7 | 18201948 | 18201972 | 25 |
| HDAC9 | 7 | 18869084 | 18869171 | 88 |
| HDAC9 | 7 | 18767203 | 18767380 | 178 |
| HDAC9 | 7 | 18914101 | 18914219 | 119 |
| HDAC9 | 7 | 18868784 | 18868839 | 56 |
| HDAC9 | 7 | 18687408 | 18687621 | 214 |
| PTEN | 10 | 89717610 | 89717776 | 167 |
| PTEN | 10 | 89690803 | 89690846 | 44 |
| PTEN | 10 | 89624227 | 89624305 | 79 |
| PTEN | 10 | 89685270 | 89685314 | 45 |
| PTEN | 10 | 89725044 | 89725226 | 183 |
| PTEN | 10 | 89692770 | 89693008 | 239 |
| PTEN | 10 | 89720651 | 89720875 | 225 |
| PTEN | 10 | 89653782 | 89653866 | 85 |
| PTEN | 10 | 89711875 | 89712016 | 142 |
| TP53 | 17 | 7572930 | 7573008 | 79 |
| TP53 | 17 | 7577499 | 7577608 | 110 |
| TP53 | 17 | 7573927 | 7574033 | 107 |
| CHD4 | 12 | 6690815 | 6690980 | 166 |
| CHD4 | 12 | 6710092 | 6710219 | 128 |
| CHD4 | 12 | 6697464 | 6697588 | 125 |
| CHD4 | 12 | 6715440 | 6715539 | 100 |
| CHD4 | 12 | 6688012 | 6688083 | 72 |
| CHD4 | 12 | 6682240 | 6682435 | 196 |
| CHD4 | 12 | 6705172 | 6705303 | 132 |
| CHD4 | 12 | 6708939 | 6709178 | 240 |
| CHD4 | 12 | 6703625 | 6703816 | 192 |
| CHD4 | 12 | 6687575 | 6687712 | 138 |
| CHD4 | 12 | 6704500 | 6704596 | 97 |
| CHD4 | 12 | 6691303 | 6691447 | 145 |
| SETD2 | 3 | 47158113 | 47158244 | 132 |
| SETD2 | 3 | 47079156 | 47079267 | 112 |
| SETD2 | 3 | 47139445 | 47139571 | 127 |
| SETD2 | 3 | 47058586 | 47058744 | 159 |
| SETD2 | 3 | 47168138 | 47168153 | 16 |
| SETD2 | 3 | 47087977 | 47088111 | 135 |
| SETD2 | 3 | 47147487 | 47147610 | 124 |
| SETD2 | 3 | 47144836 | 47144913 | 78 |
| SETD2 | 3 | 47129603 | 47129737 | 135 |
| SETD2 | 3 | 47108560 | 47108608 | 49 |
| SETD2 | 3 | 47142948 | 47143045 | 98 |
| SETD2 | 3 | 47061250 | 47061330 | 81 |
| SETD2 | 3 | 47205344 | 47205414 | 71 |

| SETD2 | 3 | 47084051 | 47084190 | 140 |
| --- | --- | --- | --- | --- |
| SETD2 | 3 | 47098280 | 47098980 | 701 |
| SETD2 | 3 | 47059128 | 47059229 | 102 |
| SETD2 | 3 | 47103653 | 47103836 | 184 |
| SETD2 | 3 | 47127685 | 47127804 | 120 |
| PPP6C | 9 | 127920520 | 127920661 | 142 |
| PPP6C | 9 | 127933364 | 127933459 | 96 |
| PPP6C | 9 | 127923120 | 127923185 | 66 |
| PPP6C | 9 | 127911955 | 127912200 | 246 |
| PPP6C | 9 | 127951198 | 127951226 | 29 |
| DDX3X | X | 41206112 | 41206265 | 154 |
| DDX3X | X | 41193506 | 41193550 | 45 |
| DDX3X | X | 41198289 | 41198336 | 48 |
| DDX3X | X | 41200737 | 41200869 | 133 |
| DDX3X | X | 41202990 | 41203657 | 668 |
| DDX3X | X | 41196661 | 41196718 | 58 |
| DDX3X | X | 41202469 | 41202604 | 136 |
| RB1 | 13 | 48951054 | 48951170 | 117 |
| RB1 | 13 | 48936951 | 48937093 | 143 |
| RB1 | 13 | 48955383 | 48955579 | 197 |
| RB1 | 13 | 48923092 | 48923159 | 68 |
| RB1 | 13 | 49047496 | 49047526 | 31 |
| RB1 | 13 | 49051491 | 49051540 | 50 |
| RB1 | 13 | 48916735 | 48916850 | 116 |
| RB1 | 13 | 49033824 | 49033969 | 146 |
| RB1 | 13 | 48878049 | 48878185 | 137 |
| RB1 | 13 | 48934153 | 48934263 | 111 |
| RB1 | 13 | 48947541 | 48947628 | 88 |
| RB1 | 13 | 49030340 | 49030485 | 146 |
| RB1 | 13 | 48921961 | 48921999 | 39 |
| RB1 | 13 | 48939030 | 48939107 | 78 |
| RB1 | 13 | 49037867 | 49037971 | 105 |
| RB1 | 13 | 48941630 | 48941739 | 110 |
| RB1 | 13 | 48942663 | 48942740 | 78 |
| RB1 | 13 | 49027129 | 49027247 | 119 |
| RB1 | 13 | 49054134 | 49054204 | 71 |
| RB1 | 13 | 48881416 | 48881542 | 127 |
| RB1 | 13 | 48919216 | 48919335 | 120 |
| RB1 | 13 | 49050837 | 49050979 | 143 |
| CTNNB1 | 3 | 41277215 | 41277334 | 120 |
| CTNNB1 | 3 | 41268699 | 41268843 | 145 |
| CTNNB1 | 3 | 41265560 | 41265572 | 13 |
| CTNNB1 | 3 | 41280625 | 41280830 | 206 |
| CTNNB1 | 3 | 41279507 | 41279567 | 61 |
| CTNNB1 | 3 | 41275630 | 41275788 | 159 |
| CTNNB1 | 3 | 41265990 | 41267357 | 1368 |
| HRAS | 11 | 534212 | 534322 | 111 |
| HRAS | 11 | 532639 | 532755 | 117 |

| ARID2 | 12 | 46231085 | 46231520 | 436 |
| --- | --- | --- | --- | --- |
| ARID2 | 12 | 46123615 | 46123925 | 311 |
| ARID2 | 12 | 46230367 | 46230815 | 449 |
| ARID2 | 12 | 46285558 | 46285884 | 327 |
| CASP8 | 2 | 202137356 | 202137670 | 315 |
| CASP8 | 2 | 202131179 | 202131519 | 341 |
| CASP8 | 2 | 202141545 | 202141696 | 152 |
| CDKN2A | 9 | 21974474 | 21974831 | 358 |
| CDKN2A | 9 | 21970896 | 21971212 | 317 |
| CHD4 | 12 | 6709370 | 6709880 | 511 |
| CHD4 | 12 | 6679840 | 6680203 | 364 |
| CHD4 | 12 | 6690205 | 6690559 | 355 |
| CHD4 | 12 | 6696545 | 6697120 | 576 |
| CHD4 | 12 | 6700627 | 6701228 | 602 |
| CHD4 | 12 | 6702252 | 6702787 | 536 |
| CHD4 | 12 | 6710420 | 6711670 | 1251 |
| CHD4 | 12 | 6707055 | 6707596 | 542 |
| CHD4 | 12 | 6701554 | 6701988 | 435 |
| CHD4 | 12 | 6691776 | 6692549 | 774 |
| CHD4 | 12 | 6686946 | 6687308 | 363 |
| ARID2 | 12 | 46287220 | 46287509 | 290 |
| RB1 | 13 | 48954120 | 48954460 | 341 |
| HRAS | 11 | 533220 | 533975 | 756 |
| CTNNB1 | 3 | 41274827 | 41275363 | 537 |
| CTNNB1 | 3 | 41277820 | 41278230 | 411 |
| DDX3X | X | 41206560 | 41206974 | 415 |
| DDX3X | X | 41201743 | 41202094 | 352 |
| DDX3X | X | 41205477 | 41205880 | 404 |
| DICER1 | 14 | 95557366 | 95557707 | 342 |
| DICER1 | 14 | 95556833 | 95557005 | 173 |
| EPHB3 | 3 | 184294600 | 184295820 | 1221 |
| EPHB3 | 3 | 184298150 | 184299420 | 1271 |
| EPHB3 | 3 | 184297250 | 184297735 | 486 |
| GNA11 | 19 | 3118890 | 3119370 | 481 |
| HDAC9 | 7 | 18535880 | 18535952 | 73 |
| HDAC9 | 7 | 18993764 | 18993878 | 115 |
| HDAC9 | 7 | 18629954 | 18630114 | 161 |
| HDAC9 | 7 | 18624899 | 18625150 | 252 |
| HDAC9 | 7 | 18631134 | 18631270 | 137 |
| KIT | 4 | 55602650 | 55603000 | 351 |
| KIT | 4 | 55593379 | 55593713 | 335 |
| KIT | 4 | 55593950 | 55594305 | 356 |
| KIT | 4 | 55592018 | 55592221 | 204 |
| MAP2K2 | 19 | 4101012 | 4101283 | 272 |
| NF1 | 17 | 29683945 | 29684400 | 456 |
| NF1 | 17 | 29548863 | 29549010 | 148 |
| NF1 | 17 | 29562624 | 29563044 | 421 |
| NF1 | 17 | 29490160 | 29490430 | 271 |

| NF1 | 17 | 29663315 | 29663937 | 623 |
| --- | --- | --- | --- | --- |
| NF1 | 17 | 29664832 | 29665162 | 331 |
| NF1 | 17 | 29559713 | 29560236 | 524 |
| PPP6C | 9 | 127951836 | 127952002 | 167 |
| PPP6C | 9 | 127915807 | 127916269 | 463 |
| RB1 | 13 | 49039110 | 49039515 | 406 |
| SF3B1 | 2 | 198267275 | 198267764 | 490 |
| SF3B1 | 2 | 198266461 | 198266859 | 399 |
| SF3B1 | 2 | 198264774 | 198265163 | 390 |
| SF3B1 | 2 | 198269750 | 198270220 | 471 |
| SH3BP1 | 22 | 38038897 | 38039186 | 290 |
| SH3BP1 | 22 | 38040639 | 38040972 | 334 |
| SH3BP1 | 22 | 38043269 | 38043533 | 265 |
| TP53 | 17 | 7578172 | 7578559 | 388 |
| TP53 | 17 | 7579307 | 7579917 | 611 |
| CHCHD2 | 7 | 56174059 | 56174183 | 125 |
| RPL29 | 3 | 52029960 | 52029960 | 1 |
| RALY | 20 | 32580927 | 32581033 | 107 |
| TERT | 5 | 1295228 | 1295451 | 224 |
| RNF185 | 22 | 31556121 | 31556137 | 17 |
| RPS14 | 5 | 149829298 | 149829355 | 58 |
| PES1 | 22 | 30988178 | 30988179 | 2 |
| ZNF778 | 16 | 89284086 | 89284086 | 1 |
| KBTBD8 | 3 | 67048537 | 67048648 | 112 |
| BLCAP | 20 | 36156393 | 36156403 | 11 |
| NFKBIE | 6 | 44233400 | 44233417 | 18 |
| MRPS31 | 13 | 41345346 | 41345346 | 1 |
| NSUN6 | 10 | 18940561 | 18940601 | 41 |
| RPS27 | 1 | 153963194 | 153963239 | 46 |
| ZFX | X | 24227012 | 24227221 | 210 |
| ZFX | X | 24228275 | 24229500 | 1226 |
| ZFX | X | 24225438 | 24225597 | 160 |
